# Supplementary material for: The internal and marginal adaptation of lithium disilicate endocrowns fabricated using intra and extraoral scanners: An in-vitro study
Source: PLoS One. 2024 Apr 16;19(4):e0301361. doi: 10.1371/journal.pone.0301361 (PMC11021016; doi:10.1371/journal.pone.0301361)

PLOS ONE

The internal and marginal adaptation of lithium disilicate endocrowns fabricated using intra and extraoral scanners: An in-vitro study

--Manuscript Draft--

**Manuscript Number:**	PONE-D-23-40785
**Article Type:**	Research Article
**Full Title:**	The internal and marginal adaptation of lithium disilicate endocrowns fabricated using intra and extraoral scanners: An in-vitro study
**Short Title:**	Digitization Techniques and Adaptation of Ceramic Endocrowns
**Corresponding Author:**	Rashin Giti, DDS, MSc<br>Shiraz University of Medical Sciences Faculty of Dentistry<br>Shiraz, Fars IRAN, ISLAMIC REPUBLIC OF
**Keywords:**	dental impression technique; digital technology; endocrown; internal and marginal adaptation; lithium disilicate.
**Abstract:**	Objective: The impression technique highly influences the adaptation of ceramic restorations. Not enough information is available to compare the marginal (MF) and internal fit (IF) of endocrowns fabricated with various digitization techniques. Therefore, this in-vitro study aimed to compare the MF and IF of lithium disilicate (LDS) endocrowns fabricated through direct and indirect digital scanning methods.<br>Materials and methods: One extracted maxillary molar was used to fabricate endocrowns. The digitization of the model was performed with (G1) direct scanning (n=10) utilizing an intraoral scanner (IOS), (G2) indirectly scanning the conventional impression taken from the model using the same IOS (n=10), (G3) indirectly digitalizing the obtained impression using an extraoral scanner (EOS) (n=10), and (G4) scanning the poured cast using the same EOS (n=10). The MF and IF of the endocrowns were measured using the replica method and a digital stereomicroscope. The Kruskal-Wallis test was used to analyze data.<br>Results: The studied groups differed significantly (p<0.001). G2 (130.31±7.87 μm) and G3 (48.43±19.14 μm) showed the largest and smallest mean vertical marginal gap, respectively. G2 and G3 led to the highest and lowest internal gaps in all regions, respectively. With significant differences among the internal regions (p<0.001), the pulpal area demonstrated the most considerable misfit in all groups.<br>Conclusion: Scanning the impression using an extraoral scanner showed smaller marginal and internal gaps.
**Order of Authors:**	Marzieh Akhlaghian, DDS, MSc
	Amir-Alireza Khaledi, DDS, MSc
	Seyed Ali Mosaddad
	Sana Dabiri, DDS
	Rashin Giti, DDS, MSc
	Farhad Kadkhodae, DDS
	Shabnam Gholami, DDS
**Additional Information:**	
**Question**	**Response**
**Financial Disclosure**<br><br>Enter a financial disclosure statement that describes the sources of funding for the work included in this submission. Review the submission guidelines for detailed requirements. View published research	The author(s) received no specific funding for this work.

Powered by Editorial Manager® and ProduXion Manager® from Aries Systems Corporation

articles from *PLOS ONE* for specific examples.<br><br>This statement is required for submission and **will appear in the published article** if the submission is accepted. Please make sure it is accurate.<br><br>**Funded studies**<br>Enter a statement with the following details:<br>• Initials of the authors who received each award<br>• Grant numbers awarded to each author<br>• The full name of each funder<br>• URL of each funder website<br>• Did the sponsors or funders play any role in the study design, data collection and analysis, decision to publish, or preparation of the manuscript?<br><br>Did you receive funding for this work?	
**Competing Interests**<br><br>Use the instructions below to enter a competing interest statement for this submission. On behalf of all authors, disclose any competing interests that could be perceived to bias this work—acknowledging all financial support and any other relevant financial or non-financial competing interests.<br><br>This statement is required for submission and **will appear in the published article** if the submission is accepted. Please make sure it is accurate and that any funding sources listed in your Funding Information later in the submission form are also declared in your Financial Disclosure statement.<br><br>View published research articles from *PLOS ONE* for specific examples.	The authors have declared that no competing interests exist.

Powered by Editorial Manager® and ProduXion Manager® from Aries Systems Corporation

**NO authors have competing interests**<br><br>Enter: *The authors have declared that no competing interests exist.*<br><br>**Authors with competing interests**<br><br>Enter competing interest details beginning with this statement:<br><br>*I have read the journal's policy and the authors of this manuscript have the following competing interests: [insert competing interests here]*<br><br>* typeset	
**Ethics Statement**<br><br>Enter an ethics statement for this submission. This statement is required if the study involved:<br><br>• Human participants<br>• Human specimens or tissue<br>• Vertebrate animals or cephalopods<br>• Vertebrate embryos or tissues<br>• Field research<br><br>Write "N/A" if the submission does not require an ethics statement.<br><br>General guidance is provided below. Consult the submission guidelines for detailed instructions. **Make sure that all information entered here is included in the Methods section of the manuscript.**	N/A

**Format for specific study types**<br><br>**Human Subject Research (involving human participants and/or tissue)**<br>• Give the name of the institutional review board or ethics committee that approved the study<br>• Include the approval number and/or a statement indicating approval of this research<br>• Indicate the form of consent obtained (written/oral) or the reason that consent was not obtained (e.g. the data were analyzed anonymously)<br><br>**Animal Research (involving vertebrate animals, embryos or tissues)**<br>• Provide the name of the Institutional Animal Care and Use Committee (IACUC) or other relevant ethics board that reviewed the study protocol, and indicate whether they approved this research or granted a formal waiver of ethical approval<br>• Include an approval number if one was obtained<br>• If the study involved *non-human primates*, add *additional details* about animal welfare and steps taken to ameliorate suffering<br>• If anesthesia, euthanasia, or any kind of animal sacrifice is part of the study, include briefly which substances and/or methods were applied<br><br>**Field Research**<br><br>Include the following details if this study involves the collection of plant, animal, or other materials from a natural setting:<br>• Field permit number<br>• Name of the institution or relevant body that granted permission	
**Data Availability**<br><br>Authors are required to make all data underlying the findings described fully available, without restriction, and from the time of publication. PLOS allows rare exceptions to address legal and ethical concerns. See the PLOS Data Policy and FAQ for detailed information.	Yes - all data are fully available without restriction

A Data Availability Statement describing where the data can be found is required at submission. Your answers to this question constitute the Data Availability Statement and **will be published in the article**, if accepted.<br><br>**Important:** Stating 'data available on request from the author' is not sufficient. If your data are only available upon request, select 'No' for the first question and explain your exceptional situation in the text box.<br><br>Do the authors confirm that all data underlying the findings described in their manuscript are fully available without restriction?	
**Describe where the data may be found in full sentences. If you are copying our sample text, replace any instances of XXX with the appropriate details.**<br><br>• If the data are **held or will be held in a public repository**, include URLs, accession numbers or DOIs. If this information will only be available after acceptance, indicate this by ticking the box below. For example: *All XXX files are available from the XXX database (accession number(s) XXX, XXX.)*.<br>• If the data are all contained **within the manuscript and/or Supporting Information files**, enter the following: *All relevant data are within the manuscript and its Supporting Information files.*<br>• If neither of these applies but you are able to provide **details of access elsewhere**, with or without limitations, please do so. For example:<br><br>*Data cannot be shared publicly because of [XXX]. Data are available from the XXX Institutional Data Access / Ethics Committee (contact via XXX) for researchers who meet the criteria for access to confidential data.*<br><br>*The data underlying the results presented in the study are available from (include the name of the third party*	The data underlying the results presented in the study are available from the corresponding author.

Powered by Editorial Manager® and ProduXion Manager® from Aries Systems Corporation

and contact information or URL).<br>• This text is appropriate if the data are owned by a third party and authors do not have permission to share the data.<br><br>* typeset	
Additional data availability information:	

Manuscript Click here to access/download;Manuscript;Manuscript.docx

The internal and marginal adaptation of lithium disilicate endocrowns fabricated using intra and extraoral scanners: An in-vitro study

Running title: Digitization Techniques and Adaptation of Ceramic Endocrowns

Marzieh Akhlaghian[1], Amir-Alireza Khaledi[1], Seyed Ali Mosaddad[2], Sana Dabiri[3], Rashin Giti[1, *], Farhad Kadkhodae[3], Shabnam Gholami[3]

1. Department of Prosthodontics, School of Dentistry, Shiraz University of Medical Sciences, Shiraz, Fars, Iran.
2. Department of Conservative Dentistry and Bucofacial Prosthesis, Faculty of Odontology, Complutense University of Madrid, Madrid, Spain.
3. Student Research Committee, School of Dentistry, Shiraz University of Medical Sciences, Shiraz, Iran.

*** Corresponding Author:** Rashin Giti. Department of Prosthodontics, School of Dentistry, Shiraz University of Medical Sciences, Ghasr-e-Dasht Street, Shiraz, Fars, Iran. Email: giti_ra@sums.ac.ir. Phone: + 98 917 108 5682.

ABSTRACT Use level 1 heading (Abstract)

Objective: The impression technique highly influences the adaptation of ceramic restorations. Not enough information is available to compare the marginal (MF) and internal fit (IF) of endocrowns fabricated with various digitization techniques. Therefore, this in-vitro study aimed to compare the MF and IF of lithium disilicate (LDS) endocrowns fabricated through direct and indirect digital scanning methods.

Materials and methods: One extracted maxillary molar was used to fabricate endocrowns. The digitization of the model was performed with (G1) direct scanning (n=10) utilizing an intraoral scanner (IOS), (G2) indirectly scanning the conventional impression taken from the model using the same IOS (n=10), (G3) indirectly digitalizing the obtained impression using an extraoral scanner (EOS) (n=10), and (G4) scanning the poured cast using the same EOS (n=10). The MF and IF of the endocrowns were measured using the replica method and a digital stereomicroscope. The Kruskal-Wallis test was used to analyze data.

Results: The studied groups differed significantly ($p<0.001$). G2 (130.31±7.87 μm) and G3 (48.43±19.14 μm) showed the largest and smallest mean vertical marginal gap, respectively. G2 and G3 led to the highest and lowest internal gaps in all regions, respectively. With significant differences among the internal regions ($p<0.001$), the pulpal area demonstrated the most considerable misfit in all groups. Re-write this section, start with results and then say it is statistically different.

Conclusion: Scanning the impression using an extraoral scanner showed smaller marginal and internal gaps.

Keywords: dental impression technique; digital technology; endocrown; internal and marginal adaptation; lithium disilicate.

Introduction

References are in brackets.

Current dentistry concepts are shifting toward more minimally invasive approaches, conserving as much tooth structure as possible (1). Thus, conventional concepts of achieving resistance and retention in prosthetic designs are now being replaced by the development of adhesive dentistry principles, shedding light on the importance of preserving tooth bio-rim, bonding protocols, and biomimetic approaches (2). In reconstructing a heavily destructed tooth structure, ceramic restorations have thus been developed to serve the purpose of conservative dentistry by providing an intaglio surface that could be bonded to the remaining tooth structure (3). One of the existing ceramic restorations is endocrown, a monolithic partial restoration covering the entire occlusal surface (4), which bonds to the prepared circumferential margins and the pulp cavity of a root canal-treated tooth through its intaglio surface (5). The adhesive cementation and prepared tooth's pulpal walls provide micro- and macro-mechanical retention, respectively (6). This restoration is highly esthetic and minimally invasive, with fewer fabrication requirements than conventional crowns (7). Endocrowns are indicated in limited interocclusal space and for molars with severely curved, resorbed, short, or weak roots unsuitable for intraradicular post-fabrication (8). One ceramic material in fabricating endocrowns is lithium disilicate (LDS). It is a glass-based ceramic that can be processed using either a heat-pressed technique or milled using digital techniques (9). High fracture strength, modulus of elasticity, flexural strength, esthetic appeal, and outstanding biocompatibility features define LDS (10).

The proximity degree between the restoration (intaglio and marginal surfaces) and abutment tooth (surface and cavo-surface angles) (11) can be used to measure the internal (IF) and marginal fit (MF) of restoration (12). Because an accurate intraoral impression is required for manufacturing a well-fitting prosthesis (13), the greater the impression accuracy, the closer the restoration-abutment proximity, and the greater the IF and MF (11). The MF is the most crucial factor in establishing a prosthesis's long-term functional success rate (14). The MF is affected by the preparation and marginal design, type of restorative material, impression approach used, and fabrication technique (15). Marginal discrepancies increase cement dissolution, plaque deposition, periodontal inflammation, and recurrent caries (16), ultimately leading to restorative failure (17, 18). The probability of recurrent carries and prosthetic failure arises from a poor cement seal that allows microorganisms to enter (19). A thick layer of cement compromises the fracture resistance

of a ceramic restoration due to increased interfacial stress and polymerization shrinkage (20). Furthermore, a poor IF could increase stresses at the tooth-restoration interface and decrease fracture resistance (21). There is no agreement on the maximum allowable marginal (MG) and internal gap (IG) for clinical success; however, values ranging from 50 to 200 µm have been documented (14, 16, 22, 23).

Adhesive dentistry has been revolutionized since the development of digital dentistry. Computer-aided design and computer-aided manufacturing (CAD/CAM) technology have rapidly become popular in dentistry, providing more time-efficient and accurate impressions with superior patient comfort and the benefit of real-time visualization (8) without the drawbacks of conventional methods, including material considerations, office-laboratory transportation, disinfection requirements, adherent inaccuracies with impression techniques, trays and mixing approaches, low working time, patient distress, the disagreeable taste of the impression materials, and nausea (14). Scanning accuracy is essential for the digital workflow to digitalize the prepared tooth surfaces precisely. A digital scan with high accuracy can improve the compatibility of the fabricated prostheses, IF, MF, and clinical success rates (8). The first step in a digital process is either taking a direct digital intraoral impression using IOSs or indirectly digitizing a conventional impression/poured cast by IOSs or extraoral scanners (EOSs) (17). However, the accuracy of a digital impression can be affected by finish line position, access difficulties, periodontal status, sulcular bleeding during scanning, salivary flow rate, patient compliance, the necessity for powder application for some devices, operator's skill, and environment lightening (17, 24-26). IOS requires numerous digital datasets to be acquired and merged due to its small scanning tips. In the digital scan and the final prosthesis, this matching process inevitably adds a minor systematic error (27). Extraoral digitization is further affected by scanning errors resulting from dimensional distortion in the impression material and the produced gypsum cast (28) and integrated errors with the stone cast pouring and conventional impression taking (29). Due to a lack of available literature comparing the adaptation of endocrowns made using various digital scanning approaches, this in-vitro study aimed to determine how direct and indirect scanning techniques using IOS and EOS could affect the MF and IF of LDS endocrown restorations. According to the null hypothesis, there would be no differences in the degree of marginal and internal discrepancies between restorations fabricated using different digital approaches.

Materials and Methods

This in-vitro study was approved by the Ethical Principles and National Norms and Standards for Conducting Medical Research in Iran under identifiers IR.SUMS.DENTAL.REC.1400.075, 1401.113, and 1401.114.

Design and preparation of the tooth

One human permanent maxillary first molar, found hopeless by a periodontist due to severe periodontitis, was selected for this research. The tooth was extracted as atraumatically as possible. An ultrasonic device (Ultrawave™ XS, Ultradent Products, Inc., USA) was used to remove any remaining tissues, adhered plaque, or calculus. The tooth was inspected using a magnification loupe (3.5×) and light to ensure it was free from cracks, anatomical defects, and carious lesions. The specimen was immersed in 5.25% sodium hypochlorite (NaOCl) for 1 hour. To prevent dryness, it was then kept in normal saline until the endodontic treatment (30).

An endodontic access cavity was initially performed using a fissure diamond bur, and the pulp tissue was removed. The preparation of the pulp cavity was finalized using a round carbide bur. The crown-down method was applied to prepare the canals with rotary instruments. The root canals were finally obturated using a sealer (AH 26; Dentsply Sirona) and gutta-percha (Gutta Percha Points; Meta Biomed, South Korea) using the lateral condensation method.

The tooth was then embedded into a self-curing acrylic resin block (Ivocron, Ivoclar Vivadent AG) to a depth of 2 mm below the cemento-enamel junction (CEJ). A small tungsten carbide rotary instrument (Hager & Meisinger GmbH) was used to remove the gutta-percha from each root canal's initial 2 mm entrance. The canal orifice was then filled using a flowable composite resin (CLEARFIL MAJESTY ES Flow, Kuraray Medical, Okayama, Japan).(31) According to Figure 1A, a diamond disk (918 BF, DZ, Lemgo, Germany) at a low speed was employed to section the tooth 2 mm above the CEJ and parallel to the occlusal surface to prepare a butt-joint design (32, 33). Using a periodontal probe suggested the preparation was performed at a 4-5 mm depth of the reduced walls, measured through the access cavity. To reach a slight internal taper (8-10º), the axial walls were prepared using a round-end taper diamond bur (Hager & Meisinger GmbH). This instrument also helped flatten the pulp chamber base and remove excessive retentive areas and round internal angles (34). Smooth internal transitions in the finishing procedure were performed using a bur (Drendel + Zweiling Diamant GmbH, Germany)

with a larger diameter and a finer particle size (35). Prepared margins were polished in the polishing phase using fine diamond burs and composite polishing instruments (EVE, Ernst Vetter GmbH, Germany). 95% ethanol was used to clean the pulpal cavity. Afterward, the prepared tooth model was digitalized using direct and indirect scanning methods based on the grouping.

Fig 1. (A) Prepared and mounted tooth model for the fabrication of endocrown; **(B)** Scanned tooth via direct approach using an intraoral scanner; **(C)** Special tray for taking a conventional impression; **(D)** A fabricated and glazed endocrown; **(E)** The fabricated restoration seated on the tooth.

The required sample size was estimated, employing data from previously published investigations (5, 15). Using a statistical power analysis program (G*Power v. 3.0.10, Heinrich-Heine-University Düsseldorf, Düsseldorf, Germany), the sample size was calculated to compare research groups while taking power (80%), α (0.05), and effect size (0.6) into account. The total number of samples was rounded to 40 (ten samples in each study group) from the calculated sample size of 36.

Group 1 - Scanning the tooth with an intraoral scanner

A single operator (M.A.) made ten direct digital scans of the prepared tooth model using an intraoral scanner (3Shape TRIOS 3, Copenhagen, Denmark) (Fig. 1B). One axial wall was the starting point for scanning, continued with the occlusal surface and ended at the opposing wall. The confocal laser mechanism-based high-speed IOS (TRIOS; 3Shape) employed in this investigation did not require powder for the scanning procedure and permitted simple handling.

Group 2 - Scanning the impression with the intraoral scanner

Initially, a cold-cured custom-made tray (Tray Material, Major, Moncalieri, Italy) was fabricated with three stops at the sides of the acrylic block (Figs. 1A and C). A wax spacer was used to make a uniform 4-mm spacing to ensure optimal material thickness in the custom tray. Increasing the retention of the impression material was performed by drilling holes into each side of the tray. An adhesive (3M™ VPS Tray Adhesive, 3M Deutschland GmbH, Germany) was applied to the tray and kept dry over the counter for 12 min per the manufacturer's recommendation. Then, a conventional impression of the prepared maxillary molar was made with polyvinyl siloxane material (Bonascan; DMP Dental Industry S.A.), using heavy and light body consistencies through

a two-step impression technique. The impression tray with the impression material inside was placed on the tooth model perpendicular to its long axis. It was held under finger pressure until the impression material was set per the manufacturer's recommendations and then lifted in a single motion. The impression-taking procedure was performed at room temperature, and the clinical setting was simulated by disinfecting the obtained impression (Impresept, 3M ESPE) for 10 minutes. To optimize the visibility of the proximal and occlusal tooth surfaces, the peripheral areas of the impression were trimmed using a scalpel. Before scanning, the impression was stored for 1 hour at room temperature and then digitized using the same IOS (36). Without pouring the impression, ten scans were performed by the same operator (M.A.) (15).

Group 3 - Scanning the impression with an extraoral scanner

Afterward, the impression was digitized using an extraoral laboratory scanner (3Shape D810; 3Shape, Copenhagen, Denmark). The same operator (M.A.) repeated the digitization method ten times without pouring the impression.

Group 4 - Scanning the cast with the extraoral scanner

After 8 hours of storage, the obtained impression was poured using a type IV dental stone (Fujirock; GC Europe N.V.). According to the manufacturer's instructions, the impression was removed from the cast 45 minutes later. Following trimming and storage for 48 hours, the master cast was scanned with the same lab EOS. The scanning procedure was repeated ten times by the same operator (M.A.). Optical scans were stored as ten standard tessellation language (STL) files for each group.

Design and fabrication of endocrowns

A CAD software program (3Shape CAD Design software; 3Shape) was employed to design similar endocrowns on all virtual preparations, as demonstrated in Figure 2. For all samples, the cement spacer was fixed to be (a) 40 µm for the internal regions 1 mm below the margin and (b) no cement spacing (0 µm) for the margin itself (37). The biogeneric reference option was used to design the occlusal anatomy for all endocrowns identically, ensuring that all restorations have the same design (38). Following the designing step, a dental milling machine (DWX-50, Roland DG Corporation, Japan) was used to fabricate the wax patterns for future restorations. After fixing the

wax blank (EASY blank wax, Renfert, Germany) in the milling chamber of the five-axis milling machine used in this study, the preview window was activated to begin milling the wax discs. An axial wax sprue (length: 5 mm, diameter: 3 mm) was fixed on the wax patterns along the material flow path. Each pattern was adjusted at an angle of 60º—the same practitioner (M.A.) designed and fabricated the wax patterns for standardization. Why restorations were not milled directly?

Fig 2. Designing the endocrown with the laboratory CAD software.

The wax patterns were invested using IPS PressVest (Ivoclar, Schaan, Liechtenstein) as a special investment material for IPS e.max. The investment ring was first pre-heated at 250 °C for half an hour without the ingot inside. Once further, the temperature elevated to 1100 degrees Celsius and held there for an hour. In this stage, a mold ready for pressing was obtained by removing the wax from the sprue channel. At 1100 °C, the placed ingot (IPS e.max Press, A2, low translucency, Ivoclar Vivadent) started plasticizing and was pressed at 3.5 bars inside the investment mold. After completing the program, the investment ring was removed from the furnace and cooled at room temperature for approximately 1 hour. A disc was then used to separate the ring. LDS fluorescent glaze paste (IPS Ivocolor glaze paste, Ivoclar Vivadent, Zürich, Switzerland) was employed to glaze the restorations. One skilled technician handled all laboratory procedures. The glazed endocrowns (Fig. 1D) were examined for imperfections, including deformation, crack, or apparent marginal misfit, and were rejected in case of deficiency. The accuracy of marginal adaptation was confirmed in case the explorer could not enter between tooth margin and restorations, and two unbiased observers confirmed the passive fit. To assure the absence of internal interference with the proper seating of restorations, a fit checker spray (Arti-Spray, BK 285, Dr. Jean Bausch, Cologne, Germany) was employed to examine the IF of all endocrowns on the tooth model. The faulty restorations underwent no adjustments, and the procedures were repeated to prepare and substitute the rejected specimens. How many restorations were found faulty and repeated per group?

Measurement procedures

Each endocrown was seated on the tooth and held under finger pressure (Fig. 1E). Images were taken with a digital camera attached to a microscope (AM413Fit Dino-Lite Pro; Dino-Lite electronic crop, Taipei, Taiwan). After connecting this microscope to a PC, the images were taken at a magnification of 80× (Fig. 3). The measurement software was first calibrated by taking a

digital photograph from a definite caliper distance at the same magnification and periodically measuring the distance of the caliper. Eight points were highlighted on the tooth 3 mm below the margin to measure the vertical MG at 45-degree intervals. Following that, photographs were captured from the explicitly designated points for each endocrown sitting. These pictures were analyzed using image analysis software (Dino Capture 2.0, AnMo Electronics Crop, Tainan Hsien, Taiwan). Using literature, the number of measures/samples was calculated (39, 40). This method made eight measurements on each sample, totaling 320 measurements across four groups (4 groups×10 samples×8 measurements). The MG of every point represented the vertical marginal misfit (µm).

Fig 3. Microscope image of a restoration-tooth interface at 80× magnification. **(a)** Lithium disilicate endocrown; **(b)** Vertical marginal gap; **(c)** Prepared tooth.

The IF and horizontal MF of the endocrowns were evaluated using the replica technique. The intaglio surface of each endocrown, coated with a light-body polyvinyl siloxane (Elite HD+; Light body-normal set, Zhermack, Italy), was placed on the tooth model mounted on a holding device. Following setting the material for 5 minutes and delicate removal of the restoration, the thin light-body layer adhered to the tooth surface was stabilized by adding a layer of regular-body material (Elite HD+; Tray material Regular body-normal set, Zhermack, Italy). Both the attached layers were removed from the model after the complete setting. Each replica was divided from the middle into four pieces along the buccolingual and mesiodistal directions. A fragment with 2 mm thickness and parallel walls was sliced from each sample to acquire a direct view from the stereomicroscope (Fig. 4). Similar segmentations and sectioning slices were applied to each replica, enabling a perpendicular viewing from the stereomicroscope platform (31).

Fig 4. Microscope image of the replica technique. **(a)** Light-body layer representing internal adaptation; **(b)** Regular-body layer.

In line with the literature, each slice was divided into four areas, including axial wall (A), pulpal floor (P), marginal (M), and cervical (C) regions (38, 41). According to Figure 5, each section underwent eight measurements: marginal (n=1; M1), cervical (n=2; C1 at the center and C2 on the cervical-axial angle), axial (n=3; A1-A3, which split the axial wall into three equally

sized sections), and pulpal (n=2; P1 at the axiopulpal angle and P2 at the center of the pulpal zone). M1 was defined as the distance between the external marginal line of the prepared model and the most extended point of the endocrown margin, C2 as the bisector of the angle between the axial wall and cervical area, P1 as the bisector of the angle between the axial wall and the pulpal floor and A1-A3, C1, and P2 as the perpendicular distances between the abutment tooth and the inner surface of the endocrown. M1 also denoted the horizontal MG and A1-A3, C1, C2, P1, and P2, the IF of the endocrown. This method used 1280 measurements across four groups, with 32 measurements on each sample. (4 groups×10 samples×4 pieces×8 measurements).

Fig 5. Schematic illustration of internal fit measurement points in the replica technique. **P1** and **P2**: pulpal discrepancies; **A1**, **A2**, and **A3**: axial discrepancies; **C1** and **C2**: cervical discrepancies; **M1:** horizontal marginal discrepancy.

A prosthodontist (AA.K.) blinded to the study's design performed all measures. The same operator repeated all measurements after two weeks to ensure data reliability. The mean of two measurements was used for analysis. All the data was stored as an Excel file. Analysis was performed on the discrepancy thickness at internal and marginal areas zones (31). The statistician who performed the analyses was also blinded to the study grouping. Figure 6 depicts the entire experimental procedure as a flow diagram.

Fig 6. A flowchart outlining the experimental procedures used to design this research

Statistical analysis

SPSS software (IBM SPSS Statistics 24, IBM SPSS Inc., Chicago, USA) was used to statistically analyze the data. The Shapiro-Wilk test was performed to assess the normality of the data, and the Kruskal-Wallis test was used to compare the obtained values by the effect of studied digitization methods on the MG and IG of LDS endocrowns. The statistical significance was adjusted at $P<0.05$, and the intraclass correlation coefficient (ICC) was calculated to analyze the correlation between the two measurements.

Results

For the primary and secondary measurements over two weeks, the intra-observer agreement was deemed satisfactory (ICC=0.878 to 0.926).

Table 1 presents the results obtained from the descriptive analysis of vertical MG, and Figure 7 shows the plot of each group. The Shapiro-Wilk test failed to meet the assumption of normality ($P<0.05$), and significant differences between the four groups were revealed by the Kruskal-Wallis test ($P<0.001$). Scanning the impression by the IOS (G2) showed the most significant mean vertical MG (130.31±7.87 µm). Scanning of the impression by the EOS (G3) showed the smallest mean vertical MG (48.43±19.14 µm).

Fig 7. Box-plot diagram of the vertical marginal gap (µm) comparisons among the four studied groups.

There is no need for this figure since the information is already in the table.

The descriptive analysis results regarding the marginal, cervical, axial, and pulpal regions obtained from the replica technique are demonstrated in Table 2. The Shapiro-Wilk test failed to support the data's normal distribution ($P<0.05$), and the Kruskal-Wallis test demonstrated significant differences among the four groups ($P<0.001$). Scanning the impression by the IOS (G2) showed the largest IG in all the four mentioned regions. In contrast, scanning the impression by the EOS (G3) directed the smallest IG in all four internal areas. The Kruskal-Wallis test demonstrated significant differences ($P<0.001$) among the four internal regions (horizontal marginal, cervical, axial, and pulpal) studied within each group. The pulpal area showed the highest gap of the four measured regions (Table 2).

The results of the descriptive analysis of total IG (The mean calculated from cervical, axial, and pulpal values) are demonstrated in Table 3. The Shapiro-Wilk test failed to support the data's normal distribution ($P<0.05$), and the Kruskal-Wallis test demonstrated significant differences among the four groups ($P<0.001$). Scanning the impression by the IOS (G2) showed the largest total IG (133.56±3.58 µm). Scanning the impression by the EOS (G3) showed the smallest total IG (68.91±8.07 µm).

Discussion

The two main digital data acquisition techniques for fabricating ceramic restorations, including endocrowns, are indirect and direct scanning. A scannable elastomeric impression or a stone cast

model is scanned in the indirect mode. In direct intraoral digitization, the preparations are directly scanned without fabricating any impression or stone model (42, 43). The present study aimed to compare the IF and vertical MF of LDS endocrowns fabricated by different direct/indirect scanning protocols. To the authors' knowledge, this study is the first to compare these digitization methods using both IOS and EOS for fabricating LDS endocrowns. Based on the results, the four investigated groups in this study differed significantly regarding MF and IF; indirect digitization of the impression using EOS had significantly better MF and IF than other methods. Therefore, the null hypothesis was rejected.

LDS was chosen as the restorative material due to its superior mechanical attributes, including its high fracture toughness of 2-3 MPa, high flexural strength of 360–440 MPa, high resistance to thermal shocks, low thermal expansion, and crystals with minimal microcrack propagation, as well as its aesthetically pleasing qualities and bonding possibility. These features have turned LDS into the golden standard material among all glass-based ceramic restorations for the fabrication of endocrowns (44, 45). As a critical step with potential effects on the IF of endocrown restorations, the wax patterns were fabricated utilizing CAD/CAM technology to minimize human error and regulate any other potentially disruptive factors, such as the distortion of the wax pattern seen frequently in conventional methods (46, 47). Furthermore, scanning was conducted on a single tooth model for G1, a single scannable impression for G2 and G3, and a single master cast for G4 to reduce the potential for error and compare only the impacts of the scanning devices and techniques. The present research did not cement the endocrowns on the prepared tooth. The MF evaluation was therefore protected against cementation-associated factors such as luting agent viscosity, thickness, type, and seating forces while cementing restorations (48, 49).

Why not milled directly ?

Clinically-acceptable MG and IG are not unanimously addressed in the literature. Different values within the range of 500-200 μm have been recommended for IG, horizontal, and vertical MG by various investigations to be clinically acceptable (50-54). As a result, all values in the current experiment were regarded as within the permissible clinical range. The replica method and direct microscopic view, as non-destructive measurement techniques, were used to quantitatively analyze the internal and marginal accuracy, allowing for the reproducibility of assessments at various time intervals while leaving the tooth intact and evaluation of marginal adaptation at either predetermined points or the restoration's whole circumference (55, 56).

Results showed that the scanning approach had an intervening influence on the gap thickness that was statistically significant. Using 3Shape D810 EOS to indirectly digitalize the taken impression (G3) showed the best results for all misfit types. Several studies have evaluated the accuracy and precision of IOSs and EOSs to date, with results that have been quite erratic, suggesting that EOSs offer nearly comparable or greater precision (15, 57-62). The present study employed the IOS in an extraoral setting, which allowed to compare the inherent accuracy of the two scanner types. However, in a clinical context, the patient's intraoral factors may be the potential cause of the disparity (63). The 3Shape TRIOS 3 IOS was based on confocal laser technology using ultrafast optical sectioning that combines the projection of structured light and confocal microscopy. When scanning severely destructed teeth with limited geometric structure, such techniques are vulnerable to deviation pattern propagation (29). EOSs, on the other hand, benefit from having several cameras and, more significantly, multi-axis motion movements, enabling more accuracy in scanning conventional impressions/casts. The 3Shape D810 EOS acts as an active triangulation device, emitting light of various wavelengths in strip patterns reflected by structure surfaces and captured by a charge-coupled device. The superiority of the EOS mechanism can thus be used to explain the results (59). Research also suggests the higher precision of EOSs in capturing undercuts and zones with high curvatures (57). Besides, the D810 EOS line scanner technology with blue light has yielded superior results in the literature compared to other devices (64, 65). Blue light scanners use light sources with shorter wavelengths, reducing scanning errors for factors affecting the color and morphology of the scanned structure (64). While IOSs employ the best-fitting algorithm to stitch the scanned images, EOSs may efficiently reconstruct the object's shape based on the point cloud produced in the three-dimensional point coordinate system (62). EOSs automatically scan a fixed model at various angles to lower operator-associated factors, potentially influencing the scanning accuracy (66). Contrary to our findings, a few earlier investigations found statistically insignificant differences between intraoral and extraoral digitization approaches regarding the MF of restorations (23, 67, 68). However, these studies mainly focused on scanning the gypsum die with EOS/IOS rather than digitization of the taken impression. Güth et al. (69) found that the accuracy of ascertained datasets depends on the scanning system, and direct digitization does not outperform indirect digitization in any studied systems. Another study found the MF of 4-unit zirconia fixed dental prostheses (FDPs) after direct scanning to be comparable to indirect digitization (11) despite the higher accuracy of the datasets produced

by the direct digital approach (70). This indicates that the higher accuracy of the virtual model datasets did not translate into a better MF of the final restorations (71). Abduljawad and Rayyan (5) found the endocrowns fabricated by directly digitalizing the tooth using an IOS or cast digitization with IOS or EOS to yield statistically insignificant differences in the mean MG and other discrepancies at cavity walls, pulpal floor, and line angles. This dissimilarity with our findings could be related to the different study designs, measuring techniques, and employing multiple tooth samples in each group, making the standardization approach challenging. Ahrberg et al. (42) found a substantially superior MF employing IOS in the fabrication of restorations. However, they only studied scanned casts and not the impressions; they used different IOS and EOS systems compared to our study, and the material tested was zirconia frameworks veneered with LDS. The potentially-significant changes during the veneering (72) and sintering of pre-sintered zirconia (71) could affect the final dimension and density of the restoration and increase the MF.

Comparing the IOS techniques, directly scanning the tooth showed better adaptation than scanning the impression in all regions. The interval between taking the impression and scanning may introduce an extra distortion, leading to higher MG and IG (73). Besides, the scanning of the tooth and impression, as different surfaces, have different degrees of accuracy due to their distinct optical characteristics (74). However, it must be considered that in-vivo studies may lead to different results due to the presence of patient-related factors influencing the intraoral scanning procedure, such as subgingival finish lines, intraoral moisture (saliva or blood), the presence of soft tissue, the opposing dentition and neighboring teeth, movements of the patient and limited space of the oral cavity, especially in the molar area. Additionally, the majority of IOSs construct digital models using image-stitching techniques. While scanning, the handheld IOS device oscillates, necessitating frequent coordinate adjustments (75). Thus, processing and matching errors accumulate with each stitching of the acquired image, leading to image distortion and erroneous readings (76). The higher the complexity and geometry of the remaining tooth structure/preparation, the lower the accuracy of IOS in data acquisition (59, 66, 69, 77).

Studies have also demonstrated high precision of the impression scanning technique with EOSs (65, 78-81). The reason that impression scanning by EOS gave better results compared to cast scanning could be attributed to the rougher surface of the casts and additional laboratory steps involved in producing gypsum casts, including inherent errors associated with pouring the master

cast, the expansion of the dental stone, trimming effects, and time-associated cast deformation (27, 82). Scanning the impression thus eliminated error-prone extra laboratory stages. Using a similar study design, Runkel et al. (83) showed that digitizing the impression without the additional step of master cast fabrication appeared to be a practical approach for scanning short spans up to one quadrant for the following restoration fabrication processes. A similar study methodology was used by Akhlaghian et al. to assess the MF of zirconia copings made utilizing direct and indirect digitization with EOS and IOS. They came to the same conclusion as our investigation: scanning the impression with a laboratory scanner was the most effective digitization approach, producing copings with the lowest MG (15).

The pulpal floor in the current study most significantly displayed the highest gap among all evaluated groups ($P<0.05$). In line with the present research, an investigation by El Ghoul et al. on the MF and IF of LDS endocrowns reported the most prominent gap at the pulpal floor of all the study groups (31). This finding can be explained by overshooting near the edges, and the imperfect scanning of the pulpal floor due to the limited optical depth of scanners for scanning the tooth and gypsum casts (38, 41, 84). The significant differences can also be related to the surface anatomy, with the more uneven surface of the pulpal floor than that of the axial wall and the distance between the scanner tip and a deep pulp chamber, increasing the IG in the digitization of the tooth and poured casts. The restricted optical depth of the device causes blurred images from the unsmooth pulpal floor and axio-pulpal line angles, leading to lower accuracy and a higher gap in these regions (35, 85). On the other hand, in G2 and G3, as the samples were negative records of the prepared model, the pulpal floor scan could be expected to lead to restorations with better adaptability in this region. However, this outcome might have been negatively affected by the maximal ceramic thickness at the pulpal area, which experienced more significant dimensional distortion through the heat treatment process (52). In this study, heat-pressed method was employed to fabricate the restorations given that surface details of the axio-pulpal line angles and pulpal floor irregularities, such as canal orifices and remaining gutta-percha, can cause restoration overmilling in the CAD/CAM workflow due to limitations in the size of milling instruments; therefore, it could cause flat surfaces such as pulpal cavity for bonding endocrowns no to be milled precisely, which yields a higher internal gap (7, 85).

A direct comparison between different studies is limited due to the lack of a standardized methodology protocol. The results of this study might have been influenced by the in-vitro setting of the study, which was not similar to clinical settings. Thus, more realistic conclusions could be drawn from further research with an in-vivo design and a larger sample size to involve the mentioned considerations in a clinical setting.

Conclusion

The present in-vitro study is concluded as follows:

1. The marginal adaptation of all lithium disilicate endocrowns fabricated following the digitization of the master model by four digitization methods, using intra and extraoral scanners, was found to be clinically acceptable.
2. The digitization technique influences marginal and internal adaptation. Indirect digitization of the obtained impression using an extraoral scanner provided significantly smaller marginal and internal gaps than other methods.
3. The gap was maximized at the pulpal floor of all the study groups.
4. Adopting a hybrid workflow to digitalize a conventional impression can help avoid the time-consuming fabrication of casts, omit superfluous stages in the working process, overcome intraoral digitization challenges where applicable, and lower errors.

Ethical Approval

This in-vitro study was approved by the Ethical Principles and National Norms and Standards for Conducting Medical Research in Iran under identifiers IR.SUMS.REC.1400.075, 1401.113, and 1401.114.

Informed consent statement

Not applicable.

Consent for publication

Not applicable.

Availability of data and materials

The data presented in this study are available on request from the corresponding author.

Competing interests

The authors declare no conflict of interest.

Funding

The study received no funding

Authors' Contribution

Conceptualization: M.A.; **Methodology:** M.A.; **Software:** S.D., F.K., and S.G.; **Validation:** M.A.; **Formal analysis:** S.D., F.K., and S.G.; **Investigation:** M.A., A.A.K., S.A.M., S.D., and F.K.; **Resources:** M.A.; **Data curation:** M.A., S.D., F.K., and S.G.; **Writing—original draft preparation:** S.A.M.; **Writing— review and editing:** S.A.M.; **Visualization:** S.A.M., A.A.K., and R.G.; **Supervision:** M.A. and R.G.; **Project administration:** M.A.; **Funding acquisition:** M.A. All authors have reviewed and approved the final draft.

Acknowledgment

The authors thank Dr. Kamran Mirzaei from the Dental Research Development Center for the statistical analysis.

References

I think the number of references is more than usual. just use the most recent to demonstrate or to compare.

1. Foxton RM. Current perspectives on dental adhesion: (2) Concepts for operatively managing carious lesions extending into dentine using bioactive and adhesive direct restorative materials. Jpn Dent Sci Rev. 2020;56(1):208-15.
2. Zafar MS, Amin F, Fareed MA, Ghabbani H, Riaz S, Khurshid Z, et al. Biomimetic Aspects of Restorative Dentistry Biomaterials. Biomimetics (Basel). 2020;5(3).
3. Lyann SK, Takagaki T, Nikaido T, Uo M, Ikeda M, Sadr A, et al. Effect of Different Surface Treatments on the Tensile Bond Strength to Lithium Disilicate Glass Ceramics. J Adhes Dent. 2018;20(3):261-8.
4. Hajimahmoudi M, Rasaeipour S, Mroue M, Ghodsi S. Evaluation of Marginal and Internal Fit of CAD/CAM Endocrowns with Different Cavity Tapers. Int J Prosthodont. 2021.
5. Abduljawad DE, Rayyan MR. Marginal and internal fit of lithium disilicate endocrowns fabricated using conventional, digital, and combination techniques. J Esthet Restor Dent. 2022;34(4):707-14.
6. Biacchi GR, Mello B, Basting RT. The Endocrown: An Alternative Approach for Restoring Extensively Damaged Molars. J Esthet Restor Dent. 2013;25(6):383-90.
7. Amini A, Zeighami S, Ghodsi S. Comparison of Marginal and Internal Adaptation in Endocrowns Milled from Translucent Zirconia and Zirconium Lithium Silicate. Int J Dent. 2021;2021:1544067.
8. Gurpinar B, Tak O. Effect of pulp chamber depth on the accuracy of endocrown scans made with different intraoral scanners versus an industrial scanner: An in vitro study. J Prosthet Dent. 2022;127(3):430-7.
9. Bastos NA, Bitencourt SB, Carneiro RF, Ferrairo BM, Strelhow SSF, Dos Santos DM, et al. Marginal and internal adaptation of lithium disilicate partial restorations: A systematic review and meta-analysis. J Indian Prosthodont Soc. 2020;20(4):338-44.
10. Altier M, Erol F, Yıldırım G, Dalkilic E. Fracture resistance and failure modes of lithium disilicate or composite endocrowns. Niger J Clin Pract. 2018;21(7):821-6.
11. Almeida e Silva JS, Erdelt K, Edelhoff D, Araújo É, Stimmelmayr M, Vieira LC, et al. Marginal and internal fit of four-unit zirconia fixed dental prostheses based on digital and conventional impression techniques. Clin Oral Investig. 2014;18(2):515-23.
12. Holmes JR, Bayne SC, Holland GA, Sulik WD. Considerations in measurement of marginal fit. J Prosthet Dent. 1989;62(4):405-8.
13. Darwish HA, Morsi TS, El Dimeery AG. Internal fit of lithium disilicate and resin nano-ceramic endocrowns with different preparation designs. Futur Dent J. 2017;3(2):67-72.
14. Falahchai M, Babaee Hemmati Y, Neshandar Asli H, Emadi I. Marginal gap of monolithic zirconia endocrowns fabricated by using digital scanning and conventional impressions. J Prosthet Dent. 2021;125(2):325.e1-.e5.
15. Akhlaghian M, Khaledi A-A, Farzin M, Pardis S. Vertical marginal fit of zirconia copings fabricated with one direct and three indirect digital scanning techniques. J Prosthet Dent. 2020.
16. Abdel-Azim T, Rogers K, Elathamna E, Zandinejad A, Metz M, Morton D. Comparison of the marginal fit of lithium disilicate crowns fabricated with CAD/CAM technology by using conventional impressions and two intraoral digital scanners. J Prosthet Dent. 2015;114(4):554-9.
17. Zarauz C, Valverde A, Martinez-Rus F, Hassan B, Pradies G. Clinical evaluation comparing the fit of all-ceramic crowns obtained from silicone and digital intraoral impressions. Clin Oral Investig. 2016;20(4):799-806.
18. Dolev E, Bitterman Y, Meirowitz A. Comparison of marginal fit between CAD-CAM and hot-press lithium disilicate crowns. J Prosthet Dent. 2019;121(1):124-8.

19. Forberger N, Göhring TN. Influence of the type of post and core on in vitro marginal continuity, fracture resistance, and fracture mode of lithia disilicate-based all-ceramic crowns. J Prosthet Dent. 2008;100(4):264-73.
20. Zhang Y, Kim JW, Bhowmick S, Thompson VP, Rekow ED. Competition of fracture mechanisms in monolithic dental ceramics: flat model systems. Journal of Biomedical Materials Research Part B: Applied Biomaterials: An Official Journal of The Society for Biomaterials, The Japanese Society for Biomaterials, and The Australian Society for Biomaterials and the Korean Society for Biomaterials. 2009;88(2):402-11.
21. Lee JH, Son K, Lee KB. Marginal and Internal Fit of Ceramic Restorations Fabricated Using Digital Scanning and Conventional Impressions: A Clinical Study. J Clin Med. 2020;9(12).
22. ABDUO J, LYONS K, SWAIN M. Fit of zirconia fixed partial denture: a systematic review. J Oral Rehabil. 2010;37(11):866-76.
23. Goujat A, Abouelleil H, Colon P, Jeannin C, Pradelle N, Seux D, et al. Marginal and internal fit of CAD-CAM inlay/onlay restorations: A systematic review of in vitro studies. J Prosthet Dent. 2019;121(4):590-7.e3.
24. Rapone B, Palmisano C, Ferrara E, Di Venere D, Albanese G, Corsalini M. The Accuracy of Three Intraoral Scanners in the Oral Environment with and without Saliva: A Comparative Study. Appl Sci. 2020;10(21):7762.
25. Nedelcu R, Olsson P, Nyström I, Thor A. Finish line distinctness and accuracy in 7 intraoral scanners versus conventional impression: an in vitro descriptive comparison. BMC Oral Health. 2018;18(1):27.
26. Mangano FG, Hauschild U, Veronesi G, Imburgia M, Mangano C, Admakin O. Trueness and precision of 5 intraoral scanners in the impressions of single and multiple implants: a comparative in vitro study. BMC Oral Health. 2019;19(1):101.
27. Shembesh M, Ali A, Finkelman M, Weber HP, Zandparsa R. An In Vitro Comparison of the Marginal Adaptation Accuracy of CAD/CAM Restorations Using Different Impression Systems. J Prosthodont. 2017;26(7):581-6.
28. Ellakany P, Tantawi ME, Mahrous AA, Al-Harbi F. Evaluation of the Accuracy of Digital Impressions Obtained from Intraoral and Extraoral Dental Scanners with Different CAD/CAM Scanning Technologies: An In Vitro Study. J Prosthodont. 2022;31(4):314-9.
29. Rudolph H, Salmen H, Moldan M, Kuhn K, Sichwardt V, Wöstmann B, et al. Accuracy of intraoral and extraoral digital data acquisition for dental restorations. J Appl Oral Sci. 2016;24(1):85-94.
30. Saglam G, Cengiz S, Karacaer O. Marginal adaptation and fracture resistance of feldspathic and polymer-infiltrated ceramic network CAD/CAM endocrowns for maxillary premolars. Niger J Clin Pract. 2020;23(1):1.
31. El Ghoul WA, Özcan M, Ounsi H, Tohme H, Salameh Z. Effect of different CAD-CAM materials on the marginal and internal adaptation of endocrown restorations: An in vitro study. J Prosthet Dent. 2020;123(1):128-34.
32. Sudha K, Mohan TM, Aparna K, Yadav BS, Rani ES, Sowjanya DL. Comparative evaluation of microleakage of conventional crowns and endocrowns using confocal laser scanning electron microscope–An in vitro study. J Dr NTR Univ Health Sci. 2020;9(1):6.
33. Foad AM, Hamdy A, Abd el Fatah G, Aboelfadl A. Influence of CAD/CAM Material and Preparation Design on the Long-term Fracture Resistance of Endocrowns Restoring Maxillary Premolars. Braz Dent Sci. 2020;23(4):9p-p.
34. Dartora NR, Moris ICM, Poole SF, Bacchi A, Sousa-Neto MD, Silva-Sousa YT, et al. Mechanical behavior of endocrowns fabricated with different CAD-CAM ceramic systems. J Prosthet Dent. 2020.

35. Hasanzade M, Sahebi M, Zarrati S, Payaminia L, Alikhasi M. Comparative Evaluation of the Internal and Marginal Adaptations of CAD/CAM Endocrowns and Crowns Fabricated from Three Different Materials. Int J Prosthodont. 2021;34(3):341–7.
36. Sağlam G, Cengiz S, Karacaer Ö. Marginal adaptation and fracture strength of endocrowns manufactured with different restorative materials: SEM and mechanical evaluation. Microsc Res Tech. 2021;84(2):284-90.
37. Rosenstiel SF, Land MF. Contemporary fixed prosthodontics-e-book: Elsevier Health Sciences; 2015.
38. Gaintantzopoulou M, El-Damanhoury H. Effect of preparation depth on the marginal and internal adaptation of computer-aided design/computer-assisted manufacture endocrowns. Oper Dent. 2016;41(6):607-16.
39. Flügge TV, Schlager S, Nelson K, Nahles S, Metzger MC. Precision of intraoral digital dental impressions with iTero and extraoral digitization with the iTero and a model scanner. Am J Orthod Dentofacial Orthop. 2013;144(3):471-8.
40. Khaledi AAR, Vojdani M, Farzin M, Pirouzi S, Orandi S. The effect of sintering time on the marginal fit of zirconia copings. J Prosthodont. 2019;28(1):e285-e9.
41. Shin Y, Park S, Park J-W, Kim K-M, Park Y-B, Roh B-D. Evaluation of the marginal and internal discrepancies of CAD-CAM endocrowns with different cavity depths: An in vitro study. J Prosthet Dent. 2017;117(1):109-15.
42. Ahrberg D, Lauer HC, Ahrberg M, Weigl P. Evaluation of fit and efficiency of CAD/CAM fabricated all-ceramic restorations based on direct and indirect digitalization: a double-blinded, randomized clinical trial. Clin Oral Investig. 2016;20(2):291-300.
43. Rudolph H, Salmen H, Moldan M, Kuhn K, Sichwardt V, Wöstmann B, et al. Accuracy of intraoral and extraoral digital data acquisition for dental restorations. Journal of Applied Oral Science. 2016;24(1):85-94.
44. Menezes-Silva R, Espinoza CAV, Atta MT, de Lima Navarro MF, Ishikiriama SK, Mondelli RFL. Endocrown: a conservative approach. Braz Dent Sci. 2016;19(2):121-31.
45. Schaefer O, Watts DC, Sigusch BW, Kuepper H, Guentsch A. Marginal and internal fit of pressed lithium disilicate partial crowns in vitro: a three-dimensional analysis of accuracy and reproducibility. Dent Mater. 2012;28(3):320-6.
46. Zinelis S. Micro-CT evaluation of the marginal fit of different In-Ceram alumina copings. Department of Prosthodontics, School of Dentistry, National and Kapodistrian University. 2009.
47. Shamseddine L, Mortada R, Rifai K, Chidiac JJ. Marginal and internal fit of pressed ceramic crowns made from conventional and computer-aided design and computer-aided manufacturing wax patterns: An in vitro comparison. J Prosthet Dent. 2016;116(2):242-8.
48. Torabi K, Vojdani M, Giti R, Taghva M, Pardis S. The effect of various veneering techniques on the marginal fit of zirconia copings. J Adv Prosthodont. 2015;7(3):233-9.
49. Kaleli N, Saraç D. Influence of porcelain firing and cementation on the marginal adaptation of metal-ceramic restorations prepared by different methods. J Prosthet Dent. 2017;117(5):656-61.
50. Huang Z, Zhang L, Zhu J, Zhao Y, Zhang X. Clinical marginal and internal fit of crowns fabricated using different CAD/CAM technologies. J Prosthodont. 2015;24(4):291-5.
51. Re D, Cerutti F, Augusti G, Cerutti A, Augusti D. Comparison of marginal fit of Lava CAD/CAM crown-copings with two finish lines. Int J Esthet Dent. 2014;9(3).
52. Nawafleh NA, Mack F, Evans J, Mackay J, Hatamleh MM. Accuracy and reliability of methods to measure marginal adaptation of crowns and FDPs: a literature review. J Prosthodont. 2013;22(5):419-28.
53. Arcuri L, Lorenzi C, Bianchi N, Marchetti E, Barlattani A. Fit evaluation of cad/cam fabricated all-ceramic restorations based on direct and indirect digitalization in vivo: a systematic review. J Biol Regul Homeost Agents. 2019;33(3 Suppl. 1):103-11.

54. Boitelle P, Mawussi B, Tapie L, Fromentin O. A systematic review of CAD/CAM fit restoration evaluations. J Oral Rehabil. 2014;41(11):853-74.
55. Guess PC, Vagkopoulou T, Zhang Y, Wolkewitz M, Strub JR. Marginal and internal fit of heat pressed versus CAD/CAM fabricated all-ceramic onlays after exposure to thermo-mechanical fatigue. J Dent. 2014;42(2):199-209.
56. Schestatsky R, Zucuni CP, Dapieve KS, Burgo TAL, Spazzin AO, Bacchi A, et al. Microstructure, topography, surface roughness, fractal dimension, internal and marginal adaptation of pressed and milled lithium-disilicate monolithic restorations. J Prosthodont Res. 2020;64(1):12-9.
57. Flügge TV, Schlager S, Nelson K, Nahles S, Metzger MC. Precision of intraoral digital dental impressions with iTero and extraoral digitization with the iTero and a model scanner. Am J Orthod Dentofacial Orthop. 2013;144(3):471-8.
58. Lee SJ, Kim SW, Lee JJ, Cheong CW. Comparison of Intraoral and Extraoral Digital Scanners: Evaluation of Surface Topography and Precision. Dent J (Basel). 2020;8(2).
59. Shimizu S, Shinya A, Kuroda S, Gomi H. The accuracy of the CAD system using intraoral and extraoral scanners for designing of fixed dental prostheses. Dent Mater J. 2017;36(4):402-7.
60. da Costa JB, Pelogia F, Hagedorn B, Ferracane JL. Evaluation of different methods of optical impression making on the marginal gap of onlays created with CEREC 3D. Oper Dent. 2010;35(3):324-9.
61. Emara A, Sharma N, Halbeisen FS, Msallem B, Thieringer FM. Comparative Evaluation of Digitization of Diagnostic Dental Cast (Plaster) Models Using Different Scanning Technologies. Dentistry Journal. 2020;8(3):79.
62. Cui N, Wang J, Hou X, Sun S, Huang Q, Lim H-K, et al. Bias Evaluation of the Accuracy of Two Extraoral Scanners and an Intraoral Scanner Based on ADA Standards. Scanning. 2021;2021:5535403.
63. Ammoun R, Suprono MS, Goodacre CJ, Oyoyo U, Carrico CK, Kattadiyil MT. Influence of Tooth Preparation Design and Scan Angulations on the Accuracy of Two Intraoral Digital Scanners: An in Vitro Study Based on 3-Dimensional Comparisons. J Prosthodont. 2020;29(3):201-6.
64. Bernal C, de Agustina B, Marín MM, Camacho AM. Performance Evaluation of Optical Scanner Based on blue LED Structured Light. Procedia Engineering. 2013;63:591-8.
65. Jeon J-H, Kim H-Y, Kim J-H, Kim W-C. Accuracy of 3D white light scanning of abutment teeth impressions: evaluation of trueness and precision. J Adv Prosthodont. 2014;6(6):468-73.
66. Fukazawa S, Odaira C, Kondo H. Investigation of accuracy and reproducibility of abutment position by intraoral scanners. Journal of Prosthodontic Research. 2017;61(4):450-9.
67. Al Hamad KQ, Al Rashdan BA, Al Omari WM, Baba NZ. Comparison of the Fit of Lithium Disilicate Crowns made from Conventional, Digital, or Conventional/Digital Techniques. J Prosthodont. 2019;28(2):e580-e6.
68. Boeddinghaus M, Breloer ES, Rehmann P, Wöstmann B. Accuracy of single-tooth restorations based on intraoral digital and conventional impressions in patients. Clin Oral Investig. 2015;19(8):2027-34.
69. Güth J-F, Runkel C, Beuer F, Stimmelmayr M, Edelhoff D, Keul C. Accuracy of five intraoral scanners compared to indirect digitalization. Clinical Oral Investigations. 2017;21(5):1445-55.
70. Güth J-F, Keul C, Stimmelmayr M, Beuer F, Edelhoff D. Accuracy of digital models obtained by direct and indirect data capturing. Clin Oral Investig. 2013;17(4):1201-8.
71. Keul C, Stawarczyk B, Erdelt KJ, Beuer F, Edelhoff D, Güth JF. Fit of 4-unit FDPs made of zirconia and CoCr-alloy after chairside and labside digitalization--a laboratory study. Dent Mater. 2014;30(4):400-7.
72. Kohorst P, Brinkmann H, Dittmer MP, Borchers L, Stiesch M. Influence of the veneering process on the marginal fit of zirconia fixed dental prostheses. J Oral Rehabil. 2010;37(4):283-91.
73. Mounajjed R, Layton DM, Azar B. The marginal fit of E. max Press and E. max CAD lithium disilicate restorations: A critical review. Dent Mater J. 2016;35(6):835-44.

74. Pedroche LO, Bernardes SR, Leao MP, KINTOPP CCdA, Correr GM, Ornaghi BP, et al. Marginal and internal fit of zirconia copings obtained using different digital scanning methods. Braz Oral Res. 2016;30(1).
75. Motel C, Kirchner E, Adler W, Wichmann M, Matta RE. Impact of Different Scan Bodies and Scan Strategies on the Accuracy of Digital Implant Impressions Assessed with an Intraoral Scanner: An In Vitro Study. Journal of Prosthodontics. 2020;29(4):309-14.
76. Kim RJ-Y, Park J-M, Shim J-S. Accuracy of 9 intraoral scanners for complete-arch image acquisition: A qualitative and quantitative evaluation. J Prosthet Dent. 2018;120(6):895-903.e1.
77. Nedelcu RG, Persson AS. Scanning accuracy and precision in 4 intraoral scanners: an in vitro comparison based on 3-dimensional analysis. J Prosthet Dent. 2014;112(6):1461-71.
78. Matta RE, Adler W, Wichmann M, Heckmann SM. Accuracy of impression scanning compared with stone casts of implant impressions. J Prosthet Dent. 2017;117(4):507-12.
79. Keul C, Runkel C, Güth JF, Schubert O. Accuracy of data obtained from impression scans and cast scans using different impression materials. Int J Comput Dent. 2020;23(2):129-38.
80. Jeon J-H, Kim D-Y, Lee J-J, Kim J-H, Kim W-C. Repeatability and reproducibility of individual abutment impression, assessed with a blue light scanner. J Adv Prosthodont. 2016;8(3):214-8.
81. Jeon J-H, Lee K-T, Kim H-Y, Kim J-H, Kim W-C. White light scanner-based repeatability of 3-dimensional digitizing of silicon rubber abutment teeth impressions. J Adv Prosthodont. 2013;5(4):452-6.
82. Baldi A, Comba A, Vergano EA, Vakalis ML, Alovisi M, Pasqualini D, et al. Digital Procedures Compared to Conventional Gypsum Casts in the Manufacturing of CAD/CAM Adhesive Restorations: 3D Surface Trueness and Interfacial Adaptation Analysis. Applied Sciences. 2021;11(11):5060.
83. Runkel C, Güth JF, Erdelt K, Keul C. Digital impressions in dentistry-accuracy of impression digitalisation by desktop scanners. Clin Oral Investig. 2020;24(3):1249-57.
84. Nejatidanesh F, Shakibamehr AH, Savabi O. Comparison of marginal and internal adaptation of CAD/CAM and conventional cement retained implant-supported single crowns. Implant Dent. 2016;25(1):103-8.
85. Zimmermann M, Valcanaia A, Neiva G, Mehl A, Fasbinder D. Three-Dimensional Digital Evaluation of the Fit of Endocrowns Fabricated from Different CAD/CAM Materials. J Prosthodont. 2019;28(2):e504-e9.

Tables

Table 1. The average endocrowns' vertical marginal gap (µm) values.

Title in bold. first raw and first column are in bold also for all tables.

Study Groups	N*	Mean±SD†	Median	Minimum	Maximum
G1<br>Intraoral Scanner – Tooth Scan	10	87.09±4.97	86.89	80.38	95.58
G2<br>Intraoral Scanner – Impression Scan	10	130.31±7.87	132.20	113.81	142.37
G3<br>Extraoral Scanner - Impression Scan	10	48.43±19.14	44.13	27.40	120.55
G4<br>Extraoral Scanner - Cast Scan	10	65.91±27.06	64.11	11.84	155.11

N*: Number; SD†: Standard Deviation

Table 2. Analysis of the gap in horizontal marginal, cervical, axial, and pulpal regions (μm).

Study Groups	Study Regions	Mean±SD†	Median	Minimum	Maximum
G1 Intraoral Scanner – Tooth Scan (N*=10)	Horizontal Marginal	93.30±3.88	94.07	85.24	98.73
	Cervical	83.60±2.76	82.57	80.37	89.31
	Axial	78.77±3.21	79.05	74.46	85.64
	Pulpal	121.87±7.78	121.93	105.85	133.57
G2 Intraoral Scanner – Impression Scan (N*=10)	Horizontal Marginal	138.87±8.05	135.88	127.35	150.76
	Cervical	131.40±5.34	131.57	122.72	138.68
	Axial	128.07±3.28	127.32	124.19	134.80
	Pulpal	142.79±3.66	141.80	139.51	152.56
G3 Extraoral Scanner - Impression Scan (N*=10)	Horizontal Marginal	19.11±4.90	19.89	11.05	26.80
	Cervical	44.12±7.49	42.45	32.90	57.65
	Axial	73.35±10.57	70.96	53.07	89.83
	Pulpal	111.97±17.20	114.65	85.27	136.38
G4 Extraoral Scanner - Cast Scan (N*=10)	Horizontal marginal	31.13±5.18	31.29	23.31	43.20
	Cervical	58.86±6.61	59.86	46.07	67.88
	Axial	87.44±10.75	89.45	67.19	101.29
	Pulpal	95.67±15.68	92.50	68.88	119.54

N*: Number; SD†: Standard Deviation

Table 3. The average values of the fabricated endocrowns' total internal gap (µm).

Study Groups	N*	Mean±SD†	Median	Minimum	Maximum
G1<br>Intraoral Scanner – Tooth Scan	10	92.57±2.12	92.29	90.22	97.41
G2<br>Intraoral Scanner – Impression Scan	10	133.56±3.58	133.22	129.81	139.98
G3<br>Extraoral Scanner - Impression Scan	10	68.91 ±8.07	71.51	56.61	78.83
G4<br>Extraoral Scanner - Cast Scan	10	75.32±7.87	78.40	59.19	82.18

N*: Number; SD†: Standard Deviation

Figure 1 Click here to access/download;Figure;Figure 1.jpg

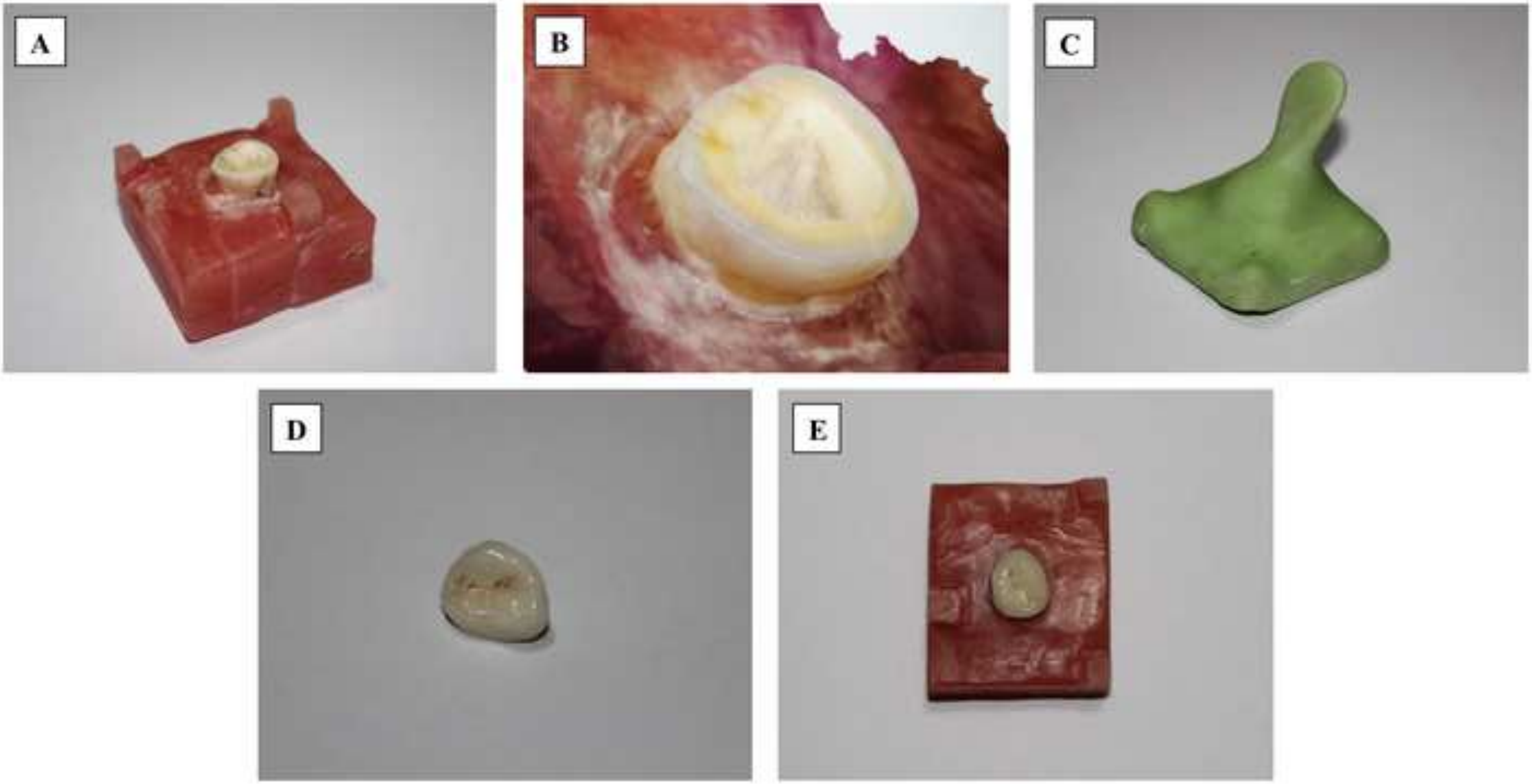

Figure 2

Click here to access/download;Figure;Figure 2.jpg

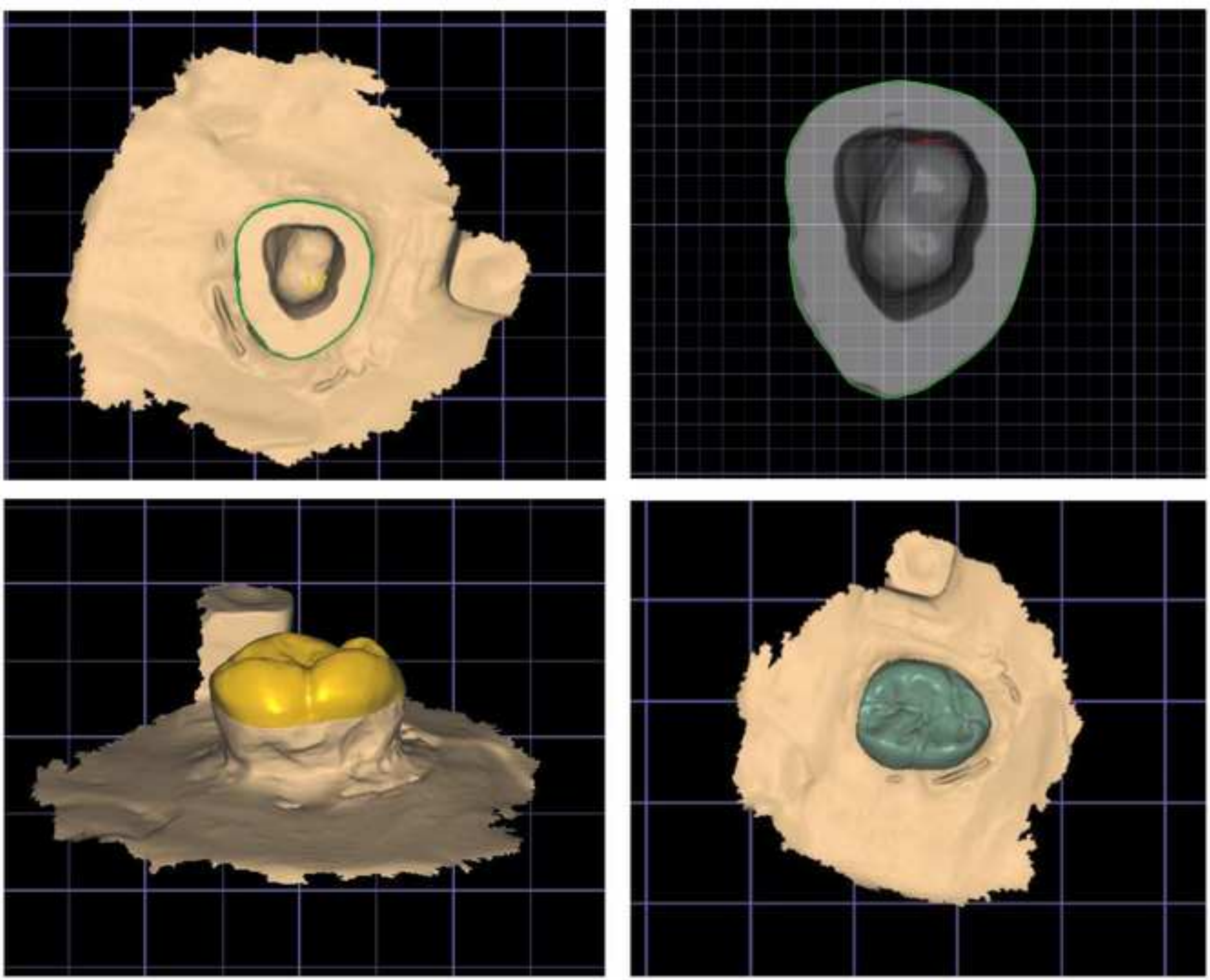

Figure 3 Click here to access/download;Figure;Figure 3.jpg

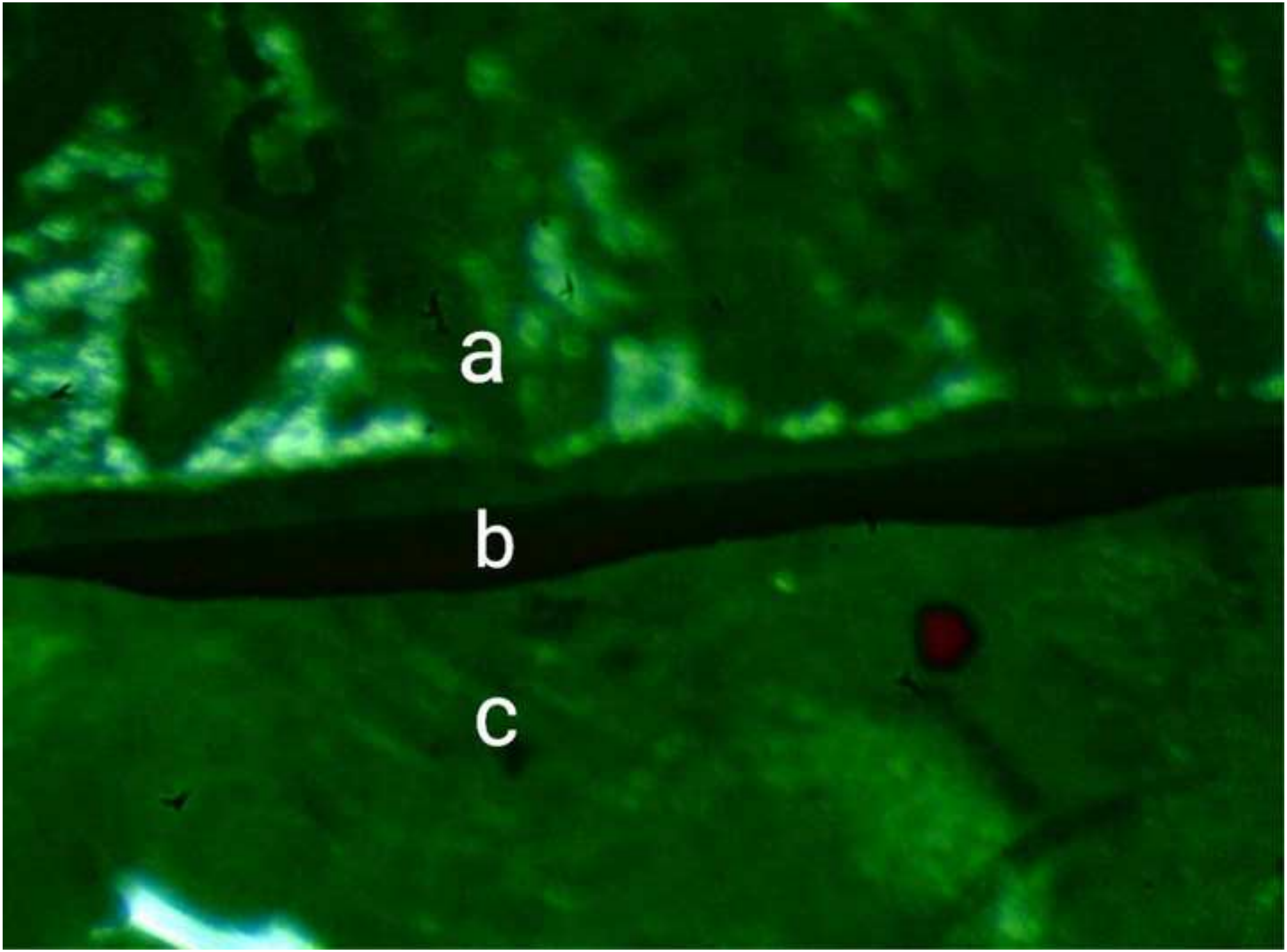

Figure 4

Click here to access/download;Figure;Figure 4.jpg

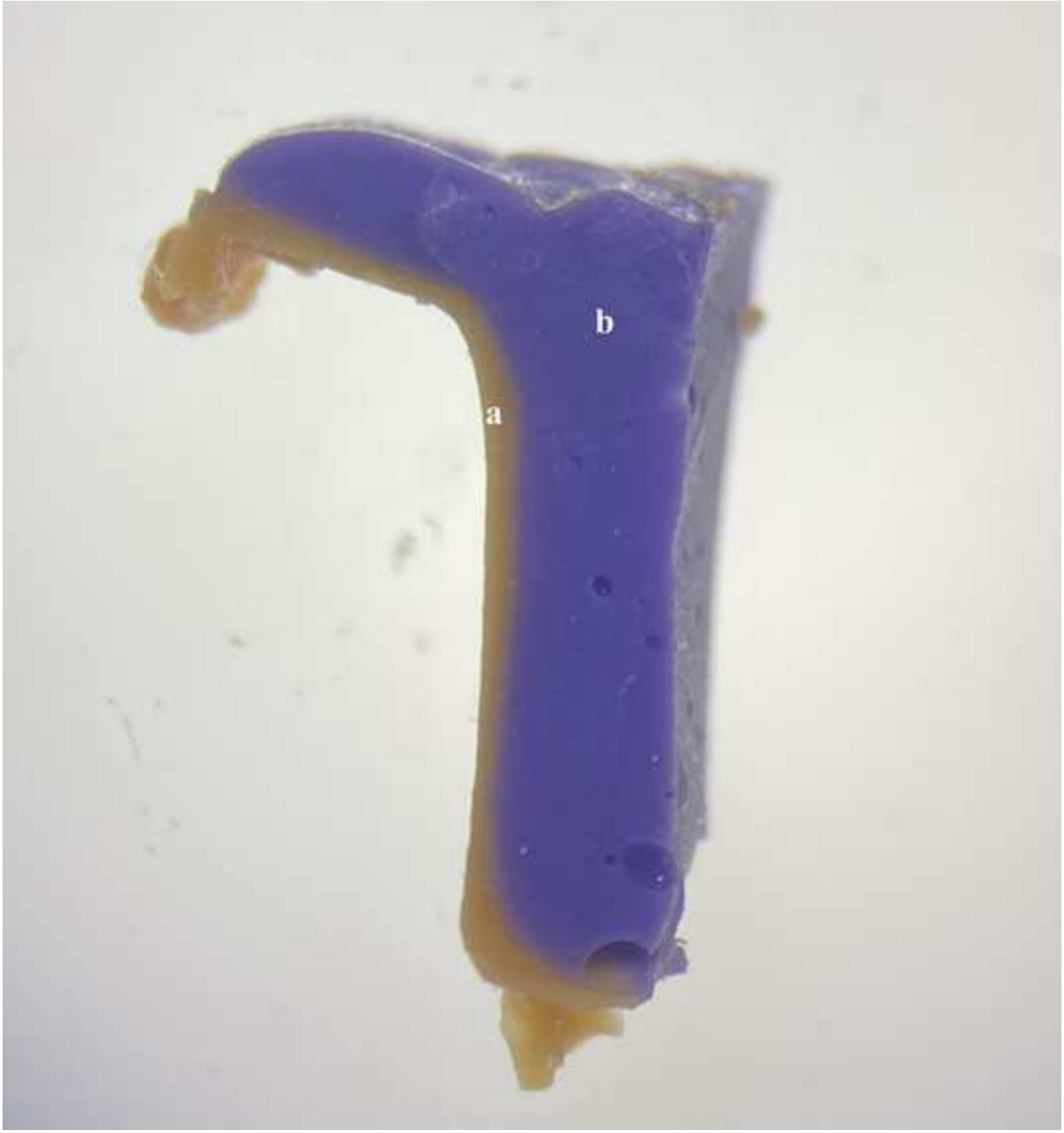

Figure 5

Click here to access/download;Figure;Figure 5.jpg

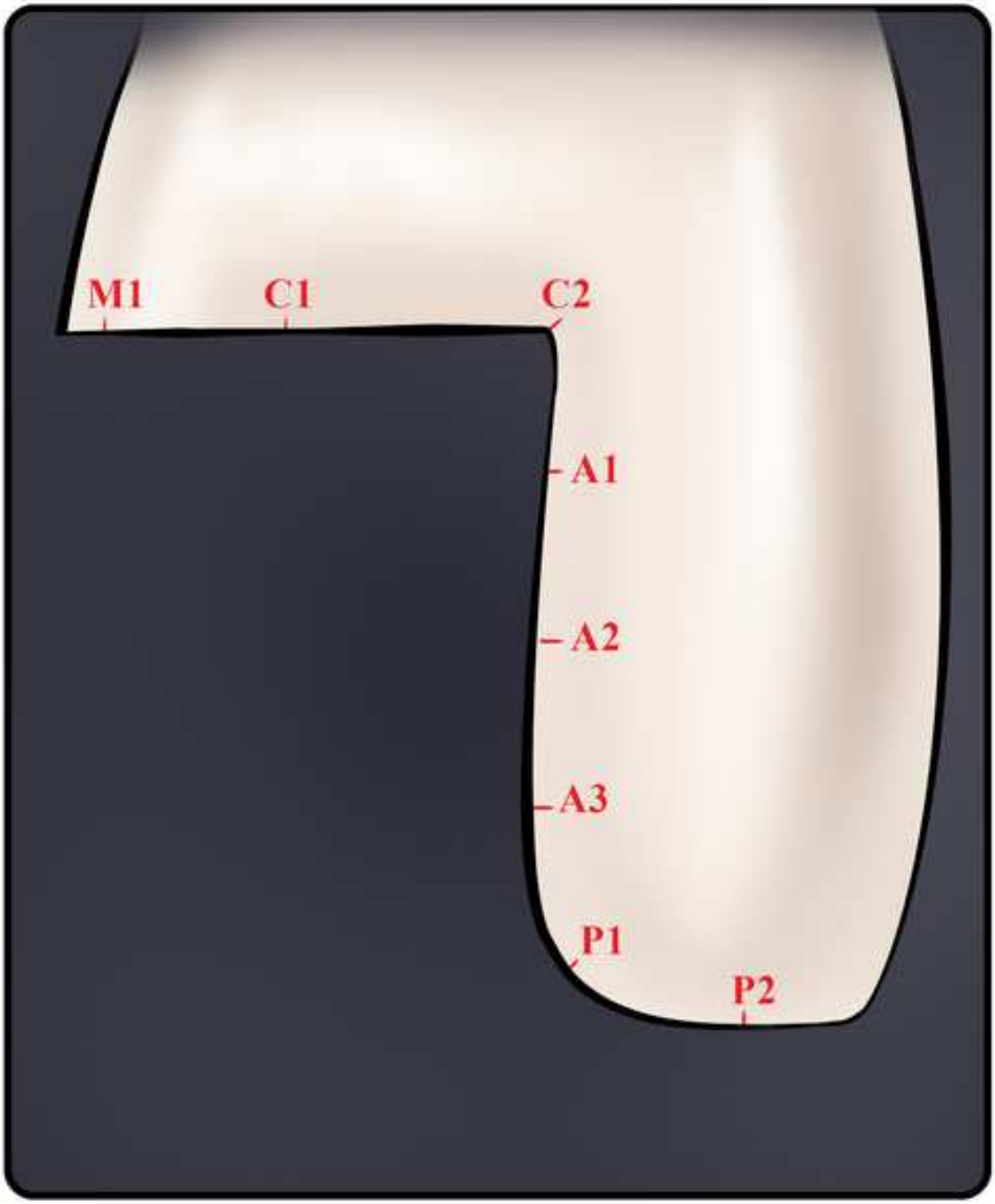

Figure 6

Click here to access/download;Figure;Figure 6.jpg

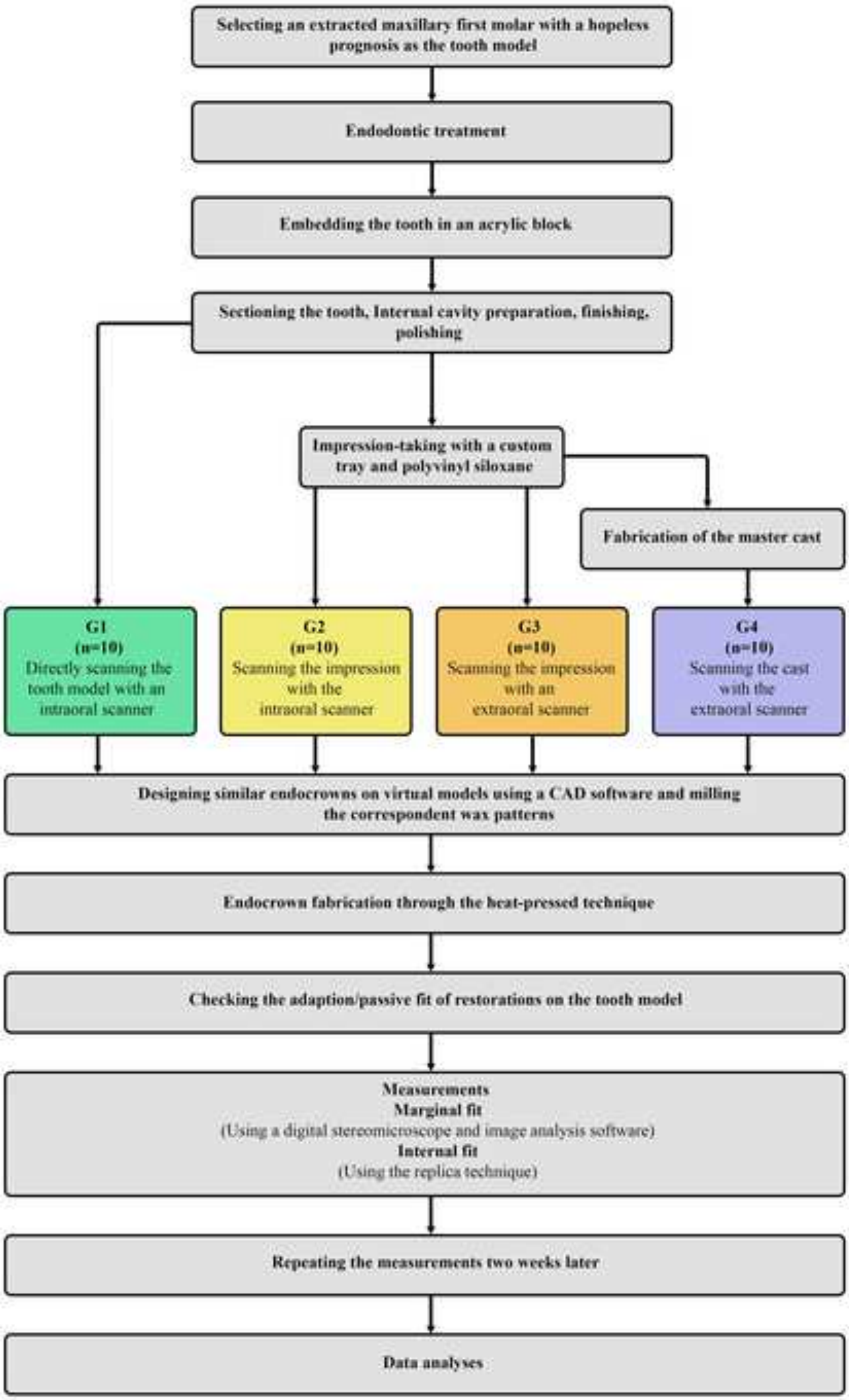

Figure 7

Click here to access/download;Figure;Figure 7.jpg

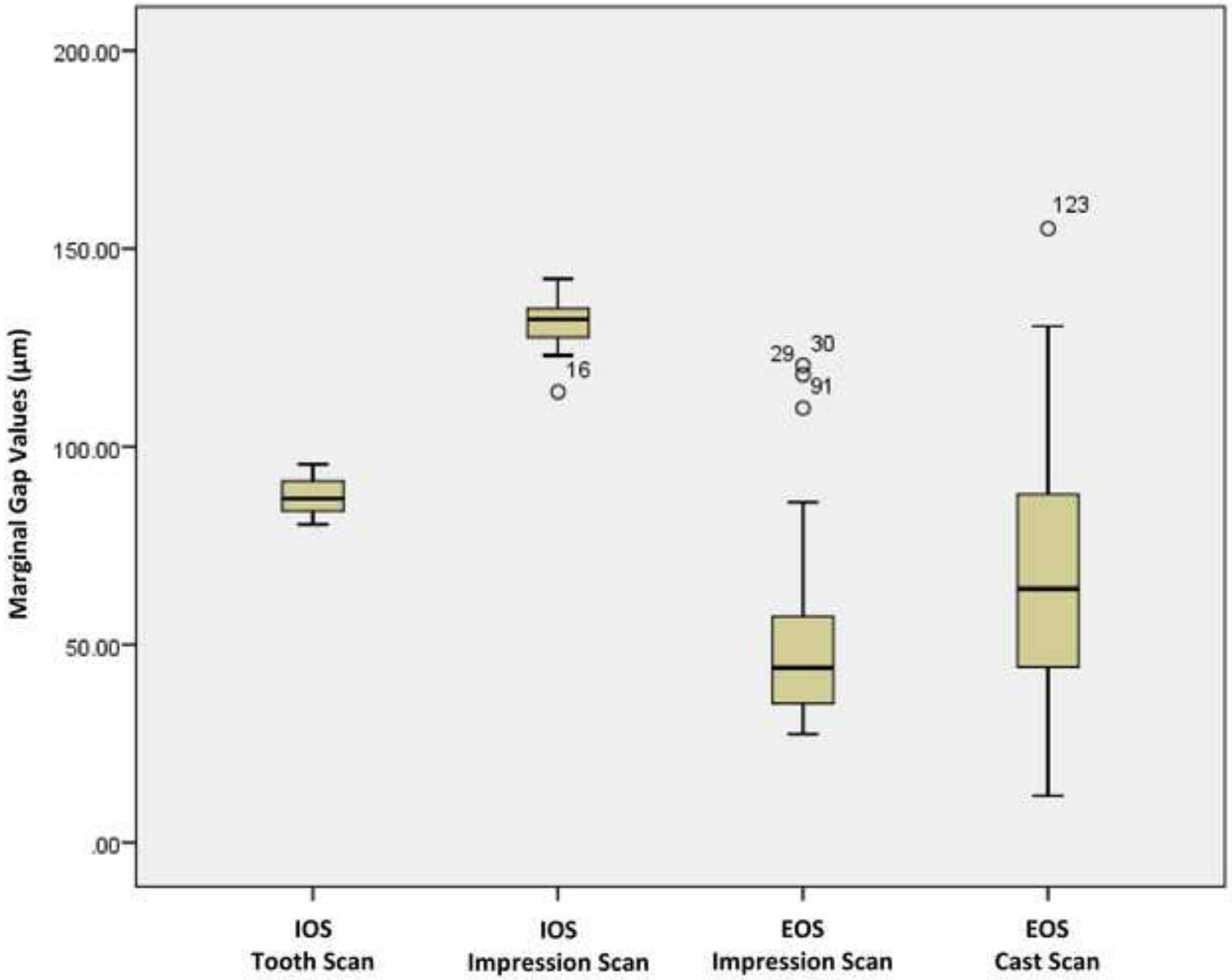

Supplement: Attachment — Submitted filename: PONE-D-23-40785.pdf [file pone.0301361.s002.pdf]
