# Supplementary material for: The internal and marginal adaptation of lithium disilicate endocrowns fabricated using intra and extraoral scanners: An in-vitro study
Source: PLoS One. 2024 Apr 16;19(4):e0301361. doi: 10.1371/journal.pone.0301361 (PMC11021016; doi:10.1371/journal.pone.0301361)
Supplement: Attachment — Submitted filename: Response to Reviewers.docx [file pone.0301361.s003.docx]

**Dear Editor and Reviewers,**

We appreciate your precious time in reviewing our paper and providing valuable comments. Your insightful words led to possible improvements in the current version. We have carefully considered the statements and tried our best to address every one of them. Besides, the entire text has been thoroughly revised to rectify English writing mistakes. We hope the manuscript, after careful revisions, meets your high standards. The authors welcome further constructive comments, if any. We provide point-by-point responses as follows.

**Reviewer #1**: The paper is well written. few formatting issues like titles of figures and tables. more important, you need to justify why you did not mill the restoration directly. why did you use the wax pattern to fabricate the restorations?

**RESPONSE:** Thank you for your valuable comment. We have modified the in-text citations of the figures to accord with the journal's guidelines. Furthermore, we have revised the figure and table caption to ensure clarity and correctness. Regarding the fabrication method, we have added to the discussion the reason we opted for the heat-pressed technique rather than milling restoration and why we used milling the wax pattern in the heat-pressed technique rather than going through the conventional wax-up process.

**Reviewer #2:** Briefly mention the limitations of the in-vitro setting and the need for further in-vivo research.

**RESPONSE:** We have dedicated a separate section to the limitations of the in vitro setting and the justification of the need to conduct more clinical studies. Thank you.

Consider quantifying the "significantly smaller" gap achieved with the optimal method for additional clarity.

**RESPONSE:** We appreciate this observation. We have revised this statement in the background to ensure it has enough clarity for the readers. Since this is the conclusion section, we avoided providing numerical data to support our statement. However, we have added that this smaller gap was in comparison to other techniques employed in this study. Additionally, the statistical data is sufficiently provided in the result section and further discussed in the discussion section.

You could optionally elaborate on the potential benefits of the proposed hybrid workflow beyond reducing errors.

**RESPONSE:** Thank you for this great feedback. We have expanded the corresponding section in the conclusion to state the benefits of the hybrid approach further.

Overall, this conclusion effectively communicates the study's contribution to the field and paves the way for further research and development.

**RESPONSE:** Thank you very much for your time and consideration in reviewing this manuscript.
